# Potential Regulation of the Long Non-Coding RNA Metastasis-Associated Lung Adenocarcinoma Transcript1 by Estrogen in Parkinson’s Disease

**DOI:** 10.3390/life14121662

**Published:** 2024-12-16

**Authors:** Eman Adel, Maya Nicolas

**Affiliations:** 1 Department of Biotechnology, School of Science and Engineering, American University in Cairo, New Cairo 11835, Egypt; emanadel@aucegypt.edu; 2 Institute of Global Health and Human Ecology, School of Science and Engineering, American University in Cairo, AUC Avenue, New Cairo 11835, Egypt

**Keywords:** Parkinson’s disease, long non-coding RNA, MALAT1, estrogen

## Abstract

Parkinson’s disease (PD) is the second-leading cause of death among neurodegenerative disease after Alzheimer’s disease (AD), affecting around 2% of the population. It is expected that the incidence of PD will exceed 12 million by 2040. Meanwhile, there is a recognized difference in the phenotypical expression of the disease and response to treatment between men and women. Men have twice the incidence of PD compared to women, who have a late onset and worse prognosis that is usually associated with menopause. In addition, the incidence of PD in women is associated with the cumulative estrogen levels in their bodies. These differences are suggested to be due to the protective effect of estrogen on the brain, which cannot be given in clinical practice to improve the symptoms of the disease because of its peripheral side effects, causing cancer in both males and females in addition to the feminizing effect it has on males. As PD pathophysiology involves alteration in the expression levels of multiple LncRNAs, including metastatic-associated lung adenocarcinoma transcript 1 (MALAT1), and as estrogen has been illustrated to control the expression of MALAT1 in multiple conditions, it is worth investigating the estrogen–MALAT1 interaction in Parkinson’s disease to mimic its protective effect on the brain while avoiding its peripheral side effects. The following literature review suggests the potential regulation of MALAT1 by estrogen in PD, which would enhance our understanding of the pathophysiology of the disease, improving the development of more tailored and effective treatments.

## 1. Introduction

Parkinson’s disease (PD) is an age-related neurodegenerative disease that is common among the elderly (>65 years). PD was first diagnosed by James Parkinson in 1817, and is now the most common movement disorder and the second most common neurodegenerative disease, after Alzheimer’s disease (AD) [1]. In 2022, the prevalence of PD in Egypt was 316.5 per 100,000 individuals, compared to 82.6 per 100,000 individuals in 2019, and the prevalence of PD is expected to continue increasing in the Middle East and North Africa [2,3]. PD affects the midbrain, where dopamine-producing neurons in the substantia nigra pars compacta (SNpc) degenerate [4]. It is also characterized by the misfolding of α-synuclein protein in the surviving neurons, forming Lewy bodies that usually appear in MRIs and are used in clinical diagnosis of the disease, which expand into other regions in the brain, including limbic and neocortical regions [5,6]. Other pathophysiological features of the disease include increased apoptosis, autophagy, and neuroinflammation. PD is a multifactorial disease in which 90% of cases are idiopathic, while only 15% are familial [7]. Environmental factors like exposure to insecticides, pesticides, and xenobiotic drugs can also contribute to the development of PD [8]. Symptoms of PD are divided into motor symptoms like resting tremors, bradykinesia, limb rigidity, and posture instability and non-motor symptoms like cognitive impairment, sleep disorder, and emotional and memory dysfunction [8]. Mostly, psychiatric symptoms are considered the burden that lowers patients’ quality of life [9]. The first-line treatment of PD is levodopa (L-dopa), which is a prodrug that can bypass the blood–brain barrier and is activated inside the brain into dopamine, which works as an agonist, binding to dopamine receptors in the basal ganglia. PD treatments only deal with relieving the symptoms, with no contribution to slowing the progression of the disease [4].

Moreover, gender plays an important role in the incidence and manifestation of PD, as men have twice the risk of developing PD compared to women. A prospective cohort study in Italy that followed 4341 elderly individuals with ages ranging from 65 to 84 years for 3 years found that men had a higher risk of developing PD compared to women, with an adjusted relative risk (RR) = 2.13 (95% CI = 1.11 to 4.11) [10]. Frentzel et al. also illustrated the same result in a retrospective, cross-sectional study (147 men and 79 women). There are biological differences between male and female brains on the molecular level that are thought to arise because of the role of estrogen in the brain. A study comparing male and female mice in multiple brain regions, including the frontal cortex (FC), hippocampus (HC), and substantia nigra (SN), found differential activity between the two genders. Reactive oxygen species (ROS) scavenging activity was different in all three regions between the two genders. Furthermore, in the established rotenone PD mouse model, there was a decrease in dihyroxyphenylalanine (DOPA) decarboxylase positive neurons, dopamine-producing neurons, and the number of astrocytes, and an increase in the activity of ROS-scavenging enzymes and microglial cells in both genders. However, in the SN region in male mice, microglial cell numbers decreased and there was no change in the number of astrocytes. In addition, in HC, there was no change in the levels of tumor necrosis factor-alpha (TNF-α) that was observed in FC and SN between males and females. Estrogen levels were also reduced in the HC and SN in addition to the altered expression of ER-α and ER-β receptors in the three regions between the two genders. This confirms the involvement of estrogen in PD pathophysiology and can explain the variation in the incidence and progression of PD in males and females [11].

## 2. Estrogen’s Role in PD

Estrogen is a steroidal hormone that is produced from astrocytes in multiple brain regions, including the thalamus, hypothalamus, and hippocampus [12]. There are three natural types of estrogen: estrone (E1), estradiol (E2), and estriol (E3), with estradiol being the most potent and the main form of estrogen that circulates the body of women during their reproductive years. It binds to the two types of estrogen receptors, ER-α and ER-β, that are present in multiple regions in the CNS and belong to the nuclear hormone receptor family, which has a ligand-binding domain and a DNA-binding domain [13]. E2 exerts its function via binding to ER, forming a complex that translocates to the nucleus and binds to the estrogen response element (ERE) sequence in the promoter regions of the downstream genes to alter their transcription [12,14]. In the CNS, E2 is involved in multiple functions, such as neurogenesis, synaptogenesis, and neural network connectivity [14].

### 2.1. Estrogen Has a Protective Effect Against PD

Multiple clinical and preclinical studies have illustrated the protective role of estrogen in PD. A longitudinal, observational cohort study comparing 63 male PD patients and 56 healthy controls (HCs) of the same age showed elevated blood levels of estradiol and testosterone in PD patients, with a negative association between estradiol levels and motor impairment symptoms [15]. Furthermore, when transcranial magnetic stimulation imaging of the primary motor cortex was conducted on 22 males and 19 females who were newly diagnosed with PD, and compared to 10 male and 19 female healthy individuals, it demonstrated that the female PD patients were more able to maintain physiological response to motor cortex stimulation compared to male PD patients. Also, male patients had an imbalance in the responsiveness of the motor cortex with a reduced motor threshold in the more deteriorated hemisphere compared to female patients who had a better, more focused response to the sensorimotor plasticity protocol [16]. Striatal dopamine active transporter (DAT) activity was observed to be higher in women than in men in a linear regression analysis of 152 men and 155 women de novo PD patients that was performed to assess the degree of striatal DAT activity in relation to their age. However, as women aged, DAT activity was greatly reduced in the anterior putamen and anterior and posterior caudate regions compared to men, which can be explained by the reduction in estrogen with age [17].

In a preclinical setting, a comparison between the male and female MitoPark (MP) model of PD illustrated that estrogen had a neuroprotective effect on dopamine-producing neurons in the SN. In female PD mice, there was a noticeable delay in the initiation of dopaminergic degeneration associated with a slow deterioration rate and less aggressive motor symptoms compared to male PD mice. On the other hand, both males and ovariectomized females showed a similar behavioral and pathophysiological manifestation of PD [18]. Li et al. demonstrated that estrogen was able to alleviate the motor and pathophysiological symptoms induced by rotenone in a PD rat model. It improved the rotarod test and increased the number of tyrosine hydroxylase (TH+) cells in the SN. Moreover, it cancelled rotenone-induced autophagy, especially in the old rat group. All these neuroprotective effects were cancelled after the treatment with the estrogen antagonist tamoxifen [19]. Siani et al. explored the neuroprotective effect of E2 after a nigrostriatal injection of 6-hydroxydopamine (6-OHDA) in male, female, OVX female, and OVX female mice treated with 17 β-estradiol as part of the model. In the OVX female group, the number of TH+ cells was significantly lower than in other groups, especially at days 7 and 14 post-surgery. Additionally, the astrocyte activation, indicated by increased expression of glial fibrillary acidic protein (GFAP), was significantly higher compared to the other groups. In the same study, Siani et al. showed that the expression of microglial activation markers was identified in the OVX mice every day following PD induction. Supplementation with E2 in the OVX+E2 group was reported to result in microglial polarization towards a cytoprotective phenotype, which was also observed in the female group [20]. Moreover, the comparison of a 6-OHDA-induced rat model that underwent ovariectomy (OP) with a 6-OHDA PD rat model (P) and with a control group showed that the OP group exhibited more intense deterioration in the learning and memory function, and in the motor function compared to both the P group and control group. This was also associated with a further reduction in the number of dopaminergic neurons in the SN compared to the P group. After treatment with genistein, a phytoestrogen, the learning and memory function was improved significantly in both the P and OP group, but it failed to improve the motor function of these models [21].

There are some contradictions regarding whether or not women had worse prognosis of PD. Frentzel et al. illustrated that men had worse motor progression, increased disease severity, and a higher L-dopa daily dose (LEDD). However, after matching the population by age, gender, and disease severity, there was no observed difference in the age of onset, motor symptoms, or disease severity between the two genders [22]. On the other hand, Solla et al. found that women tend to have worse clinical manifestations of the disease. In their case control study, they looked at 156 PD patients and 132 healthy individuals of the same age and found that women scored worse than men on the Unified Parkinson’s Disease Rating Scale (UPDRS) and the Non-Motor Symptoms Scale (NMSS) [23]. Since the level of estrogen fluctuates with women’s age, Chen et al. suggested that the worse prognosis of PD symptoms can be linked to menopause [18].

### 2.2. The Effect of the Cumulative Concentration of E2 in Women on PD

The overall concentration of E2 in women’s reproductive years is a factor that influences the incidence and manifestation of PD. Two prospective cohort studies assessing the effect of the reproductive period’s duration on the progression of motor symptoms illustrated that a short reproductive lifespan (<37 years) in female PD patients was significantly associated with rapid motor symptoms progression (SHR 0.964) as measured by the UPDRS III. Additionally, the number of children, early age of menarche, and delayed age of menopause were positively associated with postponed onset of symptoms in women PD patients by approximately 30 months [22,24]. Similarly, Pesce et al. showed that the number of children a woman had was associated with an increased incidence of PD. Early menopause also increased the risk of developing PD (HR = 1.81), which was then decreased upon supplementation with hormonal therapy. Moreover, women who used clomiphene for infertility problems had a higher risk of developing PD than women who never used it (HR = 1.81) [25]. A retrospective, cohort study investigated the incidence of motor symptoms in postmenopausal, drug-naïve women between two groups: the first group had low levels of estrogen during their reproductive lifespan while the second group had high levels of estrogen. The first group showed a worse manifestation of motor symptoms associated with lower levels of striatal dopamine transporter (DAT) availability in the posterior and ventral putamen compared to the second group. Additionally, the L-dopa dose was found to be higher in the first group [26].

### 2.3. Mechanisms by Which Estrogen Exerts Its Neuroprotective Effect

The neuroprotective effect of estrogen in the SN was suggested to be exerted through maintaining α-synuclein homeostasis and controlling its tetramer–monomer (T:M) ratio. In the 3k mouse model, where α-synuclein tetramerization was inhibited and there was an amplification of familial PD α-synuclein mutations, female mice exhibited a delay in motor symptoms manifestation and an increase in the number of TH+ cells compared to male mice. The delayed motor symptoms onset in female mice was associated with a higher degree of α-synuclein tetramerization and attenuated reduction in the dopaminergic and cortical neurons [27]. Yi et al. proposed that estrogen protects the dopaminergic neurons in the SN because it increases the expression of the brain-derived neurotrophic factor (*BDNF*) gene that protects calretinin (CR)-positive neurons from degeneration. CR belongs to the Ca-binding protein family, which makes dopamine neurons less vulnerable to degeneration. Estrogen also increased the expression of phosphorylated AKT (*p-AKT*) which reduces the apoptosis of DA neurons in PD mice induced by 1-methyl-4-phenyl-1,2,3,6-tetrahydropyridine (MPTP) [28]. In another MPTP-induced PD model with a knockout of ER-α (KO), and in astrocytes treated with 1-methyl-4-phenylpyridinium (MPP), E2 exerted its neuroprotective role on SN dopaminergic neurons via suppressing the pro-inflammatory pathway p38 MAP-Kinase (p38 MAPK). In KO mice, the number of TH+ cells was significantly lower than the wild-type (WT) mice, which was also accompanied by lower levels of dopamine and its metabolite DOPAC and higher levels of the monoamine oxidase-B (MAO-B). Moreover, the activity of the p38 MAPK pathway was higher in the SN of KO mouse models than in WT mice, explaining the activation of MAO-B. In MPP+ astrocytes, E2 treatment prevented the inflammation of the neurons and therefore decreased the levels of MAO-B, which was potentiated upon transfection with p38 inhibitors [29].

Despite the neuroprotective effect of estrogen, clinical and preclinical research introduced that women may suffer more from the side effects associated with the use of L-dopa, such as L-dopa-induced dyskinesia (LID), due to estrogen. In a prospective cohort study that examined the effect of uric acid (UA) basal levels in the development of L-dopa-induced dyskinesia (LID) in 78 male and 74 female de novo PD patients for 2 years, 23 males and 30 females developed LID [30]. Also, in a 6-OHDA ovariectomized rat model that developed LID, E2 treatment worsened abnormal involuntary movement (AIM) by increasing AIM duration, but did not cause motor symptoms progression [31].

### 2.4. Estrogen Role in the Treatment of PD

The neuroprotective effect of estrogen was comparable to current treatments. In a comparison between estrogen and L-dopa neuroprotective effects in an ovariectomized MPTP PD mouse model, estrogen exerted a significantly better behavioral recovery than L-dopa. This was present in the hanging test, foot printing test, rotarod, narrow beam walking test, and Morris’s water maze test. The exception was in the Y-maze test, where the estrogen recovery effect was not significantly higher than that of L-dopa. On the immunohistochemical level, estrogen restored the number of TH+ cells in the SN, reduced the levels of nitrite, lipid, acetylcholine esterase, and restored the levels of catalase in the SN and prefrontal cortex more significantly than levodopa [32]. However, the use of E2 as a treatment for PD cannot be applied in clinical practice because of its peripheral side effects, causing cancer in both men and women as well as causing feminization in male patients. Recent preclinical research was conducted to explore the use of CNS-specific estrogen-like 10b-17β-dihydroxyestra-1,4-dien-3-one (DHED), which had neuroprotective effects on the 3K mice male PD model as it enhanced motor symptoms, increased the rate of α-synuclein tetramerization in relation to the α-synuclein monomer, and enhanced the dopaminergic and cortical neurons neurite complexity [32]. In addition, a daily subcutaneous dose (50 and 100 μg/kg for 4 weeks) of DHED showed relief of PD pathophysiological features in an MPTP PD mouse model as it decreased degeneration of the dopaminergic neurons in the SN and the neural fibers in the striatum, decreased inflammation and oxidative stress, and improved motor and cognitive functions [33].

## 3. MALAT1 Role in the Pathophysiology of PD

Overall, 97% of the transcribed RNAs do not code for proteins, including microRNA (miRNA), long non-coding RNA (LncRNA), small interfering RNA (siRNA), and PIWI interacting RNA (piRNA) [34]. LncRNAs are >200 nucleotides in length and can regulate the expression of genes at the pretranscription, post-transcription, and post-translation levels. Many LncRNAs are involved in the modulation of memory, cognition, and synaptic plasticity in the CNS [35]. The expression of 87 LncRNAs was altered in the SN during PD, including MALAT1, HOTAIR, SNHG1, and p21 [35,36]. Metastasis-associated lung adenocarcinoma transcript 1 (MALAT1) is one of the most thoroughly studied LncRNAs, which was first recognized in 2003 as a prognostic factor for the survival of stage I non-small cell lung cancer patients [37]. MALAT1 contributes to synapse formation in the CNS via controlling the expression of multiple genes that are involved in this process. Despite the fact that MALAT1 is a ubiquitous, conserved LncRNA, its knockout in a mouse model did not affect viability or fertility. This can be due to the presence of other LncRNAs that compensate for its absence [38]. Its expression is upregulated in multiple cancers, including liver cancer, renal cell carcinoma, bladder carcinoma, colorectal cancer, breast cancer, and cervical cancer. Its upregulation is associated with a worse prognosis and metastasis of cancer [39,40]. In addition, the elevated expression of MALAT1 was associated with decreased viability, increased apoptosis, and an increased inflammatory reaction in ankylosing spondylitis (AS) [41]. MALAT1 is also involved in all pathological factors of PD and its upregulation is associated with a worse prognosis of the disease.

The expression of MALAT1 was three times higher in PD patients than in healthy controls (HC). Using specimens from the SN, limbic regions, and neocortical region, this elevation was recognized before the appearance of the phenotypical manifestation of the disease [6]. In addition, MALAT1 expression was significantly elevated in the platelets of PD patients in comparison to HCs, making it a promising non-invasive biomarker for early diagnosis of the disease in clinical settings [42]. Moreover, MALAT1 is associated with progression of the disease and the development of dementia. In a comparison between 288 sporadic PD patients and 196 HC, MALAT1 expression was significantly increased in PD patients and was associated with a lower Mini-Mental State Examination (MMSE) score. Furthermore, two SNPs, rs3200401 (C>T) and rs4102217 (G>C), were identified in MALAT1 in PD patients that increased the risk of developing Parkinson’s three-fold [43]. MALAT1 expression is upregulated in multiple PD mouse models and cell lines that showed how MALAT1 contributes to all of the hallmarks of the disease, including the expression and accumulation of α-synuclein, decreased levels of tyrosine kinase, enhanced apoptosis and neuroinflammation, and suppressed cell proliferation, which is summarized in Table 1 [4,7,9,35,36,44,45,46,47].

### 3.1. α-Synuclein

Geng et al., illustrated that MALAT1 enhanced the expression of α-synuclein via negatively regulating the expression of miR-23b-3p in the MN9D cell line treated with rotenone, which induced apoptosis of the dopaminergic neurons via the activation of microglia and initiation of neuroinflammation. Transfection of the cell line with miR-23b-3p mimics reduced the expression of both MALAT1 and α-synuclein, whereas MALAT1 knockdown increased the expression of miR-23b-3p and decreased the expression of α-synuclein. The microglial cells BV2 were divided into four groups, based on the solution that they were treated with. The four groups are as follows: (1) α-synuclein group (BV2 cells + α-synuclein solution), (2) Supernatant 1 (BV2 cells + MN9D overexpressing MALAT1 supernatant), (3) Supernatant 2 (BV2 cells + MN9D treated with pcDNA-MALAT1 and transfected with miR-23b-3p), and (4) supernatant 3 (BV2 cells + MN9D treated with pcDNA-MALAT1 and transfected with sh-α-synuclein supernatant). α-synuclein was phagocytosed into BV2 cells where its overexpression was only observed in the α-synuclein group and supernatant 1 group, which caused BV2 activation, initiation of an inflammatory response, and suppression of BV2 autophagy. The overexpression of α-synuclein in BV2 cells caused the apoptosis of MN9D cells after they were cocultured together. The inhibition of BV2 autophagy by 3-MA increased MN9D apoptosis, while the activation of BV2 autophagy by RAPA decreased MN9D apoptosis [44].

The MALAT1–α-synuclein axis was also a target of two neuroprotective substances: beta-asarone, an epilepsy treatment, and resveratrol, a natural substance that is known for its protective effect against neurological damage caused in AD, PD, and ischemic stroke [35,36]. Beta-asarone (10 mg/kg) improved PD symptoms and elevated the number of TH+ cells in an MPTP mouse model in addition to decreasing the apoptosis of the MPP+ SH-SY5Y cell line. These effects were exhibited through reversing the overexpression of MALAT1 which in turn downregulates α-synuclein in vivo and in vitro. Transfection with si-MALAT1 in MPP+ SH-SY5Y cells decreased the expression of α-synuclein only on the protein level, meaning that MALAT1 only inhibits the translation of α-synuclein, but not the transcription. This observation was only illustrated by Zhang et al. and thus needs further confirmation [36]. Similarly, resveratrol increased the number of TH+ cells and decreased apoptosis in an MPTP-induced mouse model and MPP-damaged SH-SY5Y cell line (resveratrol dose was 50 mg/kg and 50 mM, respectively). This was exhibited as resveratrol bound to the promoter region of MALAT1 gene, decreasing its expression and alleviating MALAT1’s downregulation of miR-129, which decreased the expression of the *SNCA* gene that codes for the α-synuclein protein. In vitro, the upregulation of MALAT1 cancelled the protective effect of resveratrol and increased apoptosis through sponging miR-129, diminishing its inhibitory effect on the *SNCA* gene [35]. In this paper, the inhibitory effect of resveratrol needed further confirmation by performing Western blot or ELISA analysis for apoptosis-related genes like *Bcl2*, *Bax*, and *Cleaved-casp-3*.

### 3.2. Apoptosis and Cell Proliferation

The alteration of MALAT1 expression in PD consequently changes essential biological processes that contribute to the apoptosis and autophagy of DA in the SN. miR-124 is one of the downregulated miRNAs in PD that, under normal conditions, exerts a neuroprotective effect by decreasing apoptosis and autophagy [5]. Liu et al. stated that MALAT1 induced apoptosis in a PD mouse model induced by MPTP and in MPP+ SH-SY5Y PD cell line. This occurred via downregulating the expression of miR-124, and the knockdown of MALAT1 reduced apoptosis of dopaminergic neurons as its downregulating effect on miR-124 was alleviated [4]. Also, Lu et al. illustrated that MALAT1 enhanced apoptosis as it epigenetically upregulated the death associated protein kinase 1 (*DAPK1*) gene through blocking miR-124, hindering its preventative effect on *DAPK1*, which is associated with the death of dopamine-producing neurons using the same PD mouse and cell line models. si-MALAT1 decreased apoptosis as it increased the expression of miR-124 and consequently decreased the expression of *DAPK1*. More importantly, the introduction of miR-124 inhibitor canceled the anti-apoptotic effect of MALAT1 knockdown [46].

In a different study, the MALAT1–miR-135b-5p–*GPNMB* axis was introduced as one of the mechanisms by which MALAT1 contributes to the apoptosis of dopaminergic neurons. Glycoprotein non-metastatic melanoma protein B (*GPNMB*) is another gene that is associated with damage of dopamine-producing neurons that is upregulated in PD. In the MPP-treated SK-N-SH and SK-N-BE cell lines, the overly expressed MALAT1 was associated with overexpression of the apoptosis-related proteins Bax and Cleaved-casp3 because of the downregulation of miR-135b-5p, which caused overexpression of the pro-apoptotic gene *GPNMB*. Transfection of the cell line with si-MALAT1 increased miR-135b-5p and decreased *GPNMB* expression, reducing apoptosis. Treatment with an inhibitor of miR-135b-5p reversed the effect of si-MALAT1 [45]. Furthermore, the upregulation of MALAT1 in MPTP-induced PD mice and the MPP+-treated MN9D cell line was associated with increased expression of leucine rich repeat kinase2 (*LRRK2*), one of the known mutated genes in both sporadic and familial PD patients which promotes the death of dopamine-producing neurons [48]. *LRRK2* was epigenetically controlled by MALAT1 because of its inhibitory effect on miR-205-5p, causing a decrease in cell proliferation and an increase in apoptosis. MALAT1 knockdown promoted cell proliferation and decreased apoptosis, as miR-205-5p was upregulated and *LRRK2* was downregulated. The decreased dopaminergic neuron death was diminished after treatment with an miR-205-5p inhibitor and pcDNA-*LRRK2* [7].

### 3.3. Neuroinflammation

Neuroinflammation is a major contributor to the development and prognosis of PD as it promotes the death of dopamine-producing neurons in the substantia nigra and further expands to other regions of the brain, worsening the motor symptoms and contributing to the non-motor, cognitive symptoms of the disease [49]. Yang et al. demonstrated that the increased serum levels of MALAT1 in sporadic PD patients compared to HCs were also positively correlated with increased serum levels of inflammatory markers, including interleukins, IL-1β and IL-6, tumor necrosis factor-alpha (TNF-α), and interferon gamma (IFN-γ) (rs = 0.552, 0.521, 0.550, and 0.43, respectively). In addition, in PC12 lipopolysaccharide (LPS)-activated cells, the upregulation of MALAT1 was positively associated with increasing the concentration and time of exposure to LPS. After the overexpression of MALAT1 by pcDNA3.1-MALAT1, the expression of the pro-inflammatory markers IL-6, iNOS (induced nitric oxide synthase) and IFN-γ increased, while the expression of the anti-inflammatory markers IL-10 and TGF-β1 decreased. This effect was canceled out upon knockdown of MALAT1, indicating that MALAT1 can be used as an effective indicator of inflammatory markers using serum samples [43].

In the MPTP-induced mouse PD model with LPS/ATP and activated BV2 and N2a microglia cell lines, the increased expression of MALAT1 was accompanied by upregulation of proinflammatory cytokines and the components of NLRP3 inflammasome (nucleotide oligomerization domain-like receptor family, pyrin domain containing 3), including NLRP3, ASC, and cleaved caspase 1 proteins, and downregulation of nuclear factor (erythroid-derived 2)-like-2 factor (Nrf2) transcript. Nrf2 is a known neuroprotective antioxidant that protects the cells against oxidative stress and toxins, via regulating the recruitment of inflammatory cells and the expression of genes like HO1, that fight against multiple oxidative factors. The knockdown of MALAT1 in vivo and in vitro exhibited an anti-inflammatory effect because of the upregulation of the *Nrf2* gene and downregulation of the enhancer of zeste homolog 2 gene (*EZH2*). This means that MALAT1 exerted its pro-inflammatory effect and activation of the NLRP3 inflammasome as it epigenetically reduced the expression of *Nrf2* by upregulating *EZH2*, which is a subunit of the polycomb repressive complex 2 (PRC2) that catalyzes the methylation of the promoter region of the *Nrf2* gene, decreasing its expression [47]. Moreover, MALAT1 was shown to upregulate the expression of the mitogen-activated protein kinase kinase kinase 3 (*MEKK3*) gene, which is known to enhance neuroinflammation in PD via sponging miR-124. The MALAT1–miR-124–*MEKK3* axis was suggested by Geng et al. to be included in the pathway by which methamphetamine or 3,4-Methylenedioxymethamphetamine (MDMA) increased the risk of PD. Methamphetamine is a hallucinogen drug that causes the death of dopamine-producing neurons in the SN. In a PD mouse model and the MPP+ SH-SY5Y cell line, MDMA enhanced the loss of dopaminergic neurons by the upregulation of MALAT1, which in turn, through the MALAT1–miR-124–*MEKK3* axis, reduced cell viability and TH expression and increased neuroinflammation and nitric oxide (NO) release. Knockdown and knockout of MALAT1, in MPP+ SH-SY5Y cells and an MPTP mouse model, respectively, increased the expression of miR-124, causing downregulation of the *MEKK3* gene which alleviated the neuroinflammatory effect of MDMA [9].

The contribution of MALAT1 to all pathophysiological hallmarks of PD makes it a promising therapeutic target to develop more effective treatments. Transplantation of dopaminergic neurons is a new clinical treatment for PD that was found to change the expression of MALAT1. After dopaminergic neuron transplantation into the 6-OHDA PD rat model, the improvement of symptoms was associated with altered expression of MALAT1. The PD rat model (Par group) was established by injecting 6-OHDA (25 mg/kg) into the striatal lesions of the right hemisphere while rats in the control group were injected with 5 mL 0.9% normal saline (Sal group). The Par group was transplanted with undifferentiated P19 cells (Par E group) or P19 cells after differentiation into dopaminergic cells (Par N group). The number of TH+ cells in the striatum tissue (ST) and SN was reduced in the Par group compared to the Sal group but, after transplantation, the number of TH+ cells increased in the ST in the Par-N group only and in the SN in both the Par-E and Par-N groups with the latter showing a higher increase in TH+ cells. Unexpectedly, MALAT1 was downregulated in the ST brain region and in the peripheral blood mononuclear group (PBMC) in the Par group compared to the Sal group and, upon transplantation, MALAT1 was upregulated in the Par-N group only [50]. The conflicting result about MALAT1 expression presented in this study needs further explanation. One possible explanation is that despite the interconnection between the ST and the SN in motor functions and involvement in PD, they do not necessarily exhibit the same changes at the molecular level. On the contrary, the molecular processes in the ST in the case of PD could compensate for any alteration in the SN through demonstrating an opposite pattern of MALAT1 expression similar to what was reported by Cabeza-Arvelaiz et al. about the effect of the upregulation of the human *SNCA* gene on the ST in a mouse model, which exerted a survival of neurons opposite to what happened in the SN [51].

## 4. Estrogen Regulates MALAT1

Estrogen is both a target and regulator of MALAT1. In mouse granulosa cells (mGC), MALAT1 controlled the synthesis of E2 via controlling the miR-205–*CREB1* axis. When si-MALAT1 was transfected into mGCs, it caused a reduction in E2 concentration and cAMP responsive element binding protein1 (*CREB1)* expression levels, which is involved in steroidal hormone production, but the expression of miR-205 was increased. Bioinformatics analysis, dual luciferase assays, and RNA pull-down assays suggested an interaction between miR-205 and MALAT1; thus, upon the knockdown of miR-205, both the E2 concentration and the CREB1 protein level were increased [52]. In addition, MALAT1 expression was upregulated in a prostate cancer (ER-β) cell line after treatment with 10 nM E2. ChIP, RNA-ChIP, and ChIRP suggested a correlation between MALAT1 and ER-β at the chromatin level. In vivo and ex vivo experiments suggested that MALAT1 recruitment to chromatin was reduced after treatment with E2, increasing the transcription of the ER-β target genes *pS2* and *PSA*. As in the absence of E2, there was an interaction between MALAT1, the DNA binding domain of ER-β, and the CB4 subunit of the polycomb repressive complex1 (PRC1) on the estrogen response element (ERE) sequence in the promoter region of ER-β target genes, favoring a closed chromatin conformation. This indicated that MALAT1 knockdown cancelled the sensitivity of ER-β target genes (*pS2* and *PSA*) to E2 as their expression increased regardless of the presence or absence of E2. This means that MALAT1 can act as a chromatin modifier, affecting the transcription of ER-β target genes in a hormone-dependent manner [53]. Du et al. demonstrated that MALAT1 was upregulated by estradiol (E2) in endometriosis. Comparing ectopic endometrium, utopic endometrium, and normal endometrium, MALAT1 was significantly upregulated in the ectopic group compared to the other two groups, which was associated with the downregulation of miR200s. When ECC cells and Ishikawa cells were treated with E2 (10^−8^ mol/L), a further elevation of MALAT1 expression and further reduction in miR200s expression were illustrated. This effect was not observed in ECC and Ishikawa cells that were treated with the ER-β antagonist PHTPP, differing from when they were treated with an ER-α antagonist. In addition, when the researchers of the paper conducted a bioinformatics analysis, it suggested the presence of ERE in the promoter region of MALAT1 providing a possible mechanism by which E2 increased the expression of MALAT1 [54].

**Figure 1:**
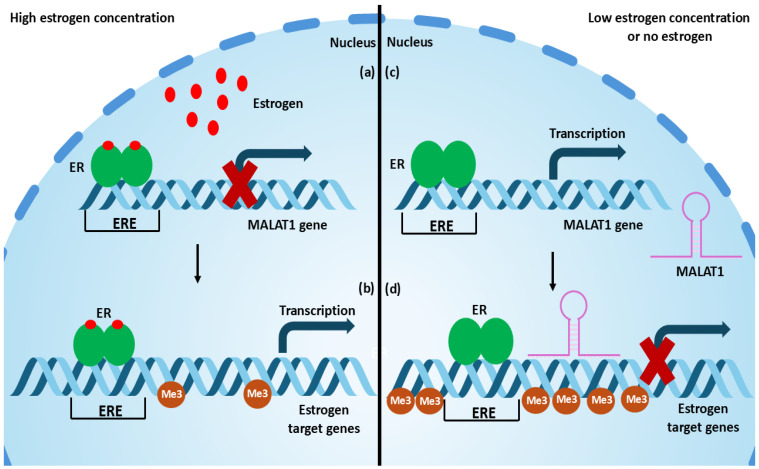
**An illustration of the potential relationship between estrogen and MALAT1 in PD**. (**a**) The inhibitory effect of high-dose estrogen on the expression of MALAT1. (**b**) The activated expression of estrogen target genes in the absence of MALAT1 through favoring open chromatin remodeling. (**c**) The stimulatory effect of low-dose or no estrogen on the expression of MALAT1. (**d**) The inhibited expression of estrogen target genes in the presence of MALAT1 through favoring a closed chromatin remodeling. ER, estrogen receptor; ERE, estrogen response element; Me3 H3K27me3, trimethylation of lysine 27 on the histone H3 protein.

Aiello et al. demonstrated that in breast cancer (ER-α), MALAT1 expression was upregulated after treatment with 10 nM E2. On the other hand, Zhao et al. introduced that MALAT1 expression in breast cancer cells expressing ER-α decreased when it was treated with high-dose E2 (100 nM for 24 h), independent from ER-α, decreasing proliferation. Knockdown of MALAT1 in these breast cancer cells also illustrated a decrease in cell proliferation, suggesting that high E2 doses reduced proliferation of breast cancer cells (ER-α) via reducing the expression of MALAT1 [55]. Moreover, MALAT1 expression was downregulated when osteosarcoma MG-63 cells were treated with a high dose of 17 β-estradiol (100 nM) for 24 h. The downregulation of MALAT1 was due to the upregulation of miR-9 induced by the treatment of E2. Moreover, miR-9 knockdown halted the downregulatory effect of E2 on MALAT1. The presence or absence of ER-α in MG-63 did not have any implications on the expression of MALAT1 or miR-9 and their downstream genes, indicating that the high-dose E2 effect on OS is independent from ER-α [56]. This means that the effect of E2 on MALAT1 expression is dose-dependent where low doses (10 nM or 10^−8^ mol/L) cause an upregulation of MALAT1 while high doses (100 nM) cause a downregulation of MALAT1, explaining the increased incidence and the worse prognosis of PD in women after menopause where E2 concentration drops dramatically. All these studies highlight the dynamic, interdependent relationship between MALAT1 and estrogen, explaining the potential mechanism by which estrogen exerts its neuroprotective effect as a means of regulating MALAT1 expression. Thus, high doses of estrogen bind to the ERE in the promoter region of MALAT1, decreasing its expression, which in turn decreases the expression of MALAT1 target genes involved in the pathophysiology of the disease while increasing the expression of estrogen target genes by promoting a more open chromatin remodeling (Figure 1).

## 5. Discussion

A reciprocal regulation between MALAT1 and E2 is suggested in the context of PD, but this hypothesis has not been tested yet. Apparently, there is a notable lack in the research that studies the relationship between MALAT1 and estrogen in general and in PD in particular. Therefore, more studies are needed to further illustrate this relationship, taking into consideration the change in MALAT1 expression levels in response to different E2 concentrations. What has been introduced in the literature is very promising, paving the way for more in silico and experimental research to test this hypothesis which would allow an efficient understanding of the disease. The use of non-human primates as an animal model to study all of the mechanisms illustrated in the literature would enhance our understanding of the topic. Moreover, the influence of gender on the molecular and phenotypical expression of PD is somehow overlooked and requires specially designed experiments that take gender differences into consideration, which is what has been recommended by the WHO. In addition, there is a gap in the literature regarding finding a link between gender and the expression level of LncRNAs, especially MALAT1, in PD. This would help in developing more customized, efficient treatments that not only alleviate the symptoms of PD, but rather work on the root cause of the disease especially for women who are at greater danger of developing treatment-induced side effects like L-dopa-induced dyskinesia [30,31]. This literature highlighted the available data about the underlying cause of the incidence difference of PD in males versus females, the contribution of MALAT1 in the pathophysiology of Parkinson’s, and the available data in the literature regarding the association between MALAT1 and estrogen.

## Figures and Tables

**Table 1:** Summary of the contribution of MALAT1 in PD pathophysiology.

Author	Year	Model	miRNA	Gene/Protein	Result
Geng et al. [44]	2023	MN9D treated with rotenone/BV2 cells.	↓miR-23b-3p	↑α-synuclein	↑Apoptosis ↑Neuroinflammation
Zhang et al. [36]	2016	MPTP-induced mouse model/MPP+ SH-SY5Y cell line.		↑α-synuclein	↑Apoptosis ↓TH+ cells
Xia et al. [35]	2019	MPTP-induced mouse model/MPTP + SH-SY5Y cell line.	↓miR-129	↑*SNCA* gene	↑Apoptosis ↓TH+ cells
Liu et al. [4]	2017	MPTP-induced mouse model/MPP+ SH-SYSY-induced cell line.	↓miR-124		↑Apoptosis
Lu et al. [46]	2020	MPTP-induced mouse model/MPP+ SH-SY5Y cell line.	↓miR-124	↑*DAPK1*	↑Apoptosis
Lv et al. [45]	2021	MPP+ SK-N-SH and SK-N-BE cell line.	↓miR-135b-5b	↑*GPNMB*	↑Apoptosis
Chen et al. [7]	2018	MPTP-induced mouse model/MPP+ MN9D cell line	↓miRNA-205-5p	↑*LRRK2*	↑Apoptosis ↓cell proliferation
Yang et al. [43]	2021	LPS + PC12 cells		↑*IL-6* ↑*iNOS* ↑*IFN-γ* ↓*IL-10* ↓*TGF-β1*	↑inflammation
Cai et al. [47]	2020	MPTP-induced mouse model/LPS/ATP activated BV2 and N2a microglia cell line.		↑*EZH2/H3K17me3* ↓*Nrf2*	↑proinflammatory cytokines. ↑NLRP3 inflammasome
Geng et al. [9]	2023	MPTP-induced mouse model/MPP+ SH-SYSY-induced cell line.	↓miR-124	↑*MEKK3*	↑Apoptosis ↑Neuroinflammation ↑NO ↓cell proliferation ↓TH+ cells

(↑) and (↓), respectively, indicate an upregulation/downregulation for the miRNA, gene, or protein and an increased/decreased level in the result column. MPTP, 1-methyl-4-phenyl-1,2,3,6-tetrahydropyridine; MPP, 1-methyl-4-phenylpyridinium; LPS, lipopolysaccharide; ATP, adenosine triphosphate; *DAPK1*, death associated protein kinase 1; *GPNMB*, Glycoprotein non-metastatic melanoma protein B; *LRRK2*, Protein rich repeat kinase 2; *IL-6*, interleukin 6; *iNOS*, induced nitric oxide synthase; *IFN-γ*, interferon-gamma; *IL-10*, interleukin 10; *TGF-β1*, Transforming growth factor beta; *NLRP3*, inflammasome nucleotide oligomerization domain-like receptor family; pyrin, domain containing 3; *Nrf2*, nuclear factor (erythroid-derived 2)-like-2 factor; *MEKK3*, mitogen-activated protein kinase kinase kinase 3; *EZH2*, Enhancer of zeste homolog 2.
